# Therapeutic Effects of Live *Lactobacillus plantarum* GKD7 in a Rat Model of Knee Osteoarthritis

**DOI:** 10.3390/nu14153170

**Published:** 2022-08-01

**Authors:** Yen-You Lin, Sunny Li-Yun Chang, Shan-Chi Liu, David Achudhan, You-Shan Tsai, Shih-Wei Lin, Yen-Lien Chen, Chin-Chu Chen, Jun-Way Chang, Yi-Chin Fong, Sung-Lin Hu, Chih-Hsin Tang

**Affiliations:** 1 Department of Pharmacology, School of Medicine, China Medical University, Taichung 404333, Taiwan; chas6119@gmail.com; 2 Graduate Institute of Biomedical Science, China Medical University, Taichung 404333, Taiwan; liyunchang@mail.cmu.edu.tw (S.L.-Y.C.); achudhaninformations@gmail.com (D.A.); 3 School of Medicine, China Medical University, Taichung 404333, Taiwan; 4 Department of Medical Education and Research, China Medical University Beigang Hospital, Yunlin 651012, Taiwan; sdsaw.tw@yahoo.com.tw; 5 Biotech Research Institute, Grape King Bio Ltd., Taoyuan 325002, Taiwan; youshan.tsai@grapeking.com.tw (Y.-S.T.); wei.lin@grapeking.com.tw (S.-W.L.); lan.chen@grapeking.com.tw (Y.-L.C.); 6 Institute of Food Science and Technology, National Taiwan University, Taipei 106617, Taiwan; gkbioeng@grapeking.com.tw; 7 Department of Food Science, Nutrition and Nutraceutical Biotechnology, Shih Chien University, Taipei 104336, Taiwan; 8 Department of Bioscience Technology, Chung Yuan Christian University, Taoyuan 320314, Taiwan; 9 The Ph.D. Program of Biotechnology and Biomedical Industry, China Medical University, Taichung 404333, Taiwan; u103003404@cmu.edu.tw; 10 Department of Sports Medicine, College of Health Care, China Medical University, Taichung 404333, Taiwan; yichin.fong@gmail.com; 11 Department of Orthopaedic Surgery, China Medical University Beigang Hospital, Yunlin 651012, Taiwan; 12 Department of Family Medicine, China Medical University Hsinchu Hospital, Hsinchu 302056, Taiwan; 13 Chinese Medicine Research Center, China Medical University, Taichung 404333, Taiwan; 14 Department of Medical Laboratory Science and Biotechnology, Asia University, Taichung 413305, Taiwan

**Keywords:** osteoarthritis, inflammation, *Lactobacillus plantarum* GKD7, IL-1β, TNF-α, anterior cruciate ligament transection

## Abstract

Osteoarthritis (OA) is a painful, progressive chronic inflammatory disease marked by cartilage destruction. Certain synovial inflammatory cytokines, such as IL-1β and TNF-α, promote OA inflammation and pain. *Lactobacillus* spp. is a well-known probiotic with anti-inflammatory, analgesic, antioxidant, and antiosteoporotic properties. This study evaluated the therapeutic effects of a live *L. plantarum* strain (GKD7) in the anterior cruciate ligament transection (ACLT)-induced OA rat model. The results show that oral administration of live *L. plantarum* GKD7 improved weight-bearing asymmetry after ACLT surgery. Moreover, micro-computed tomography images and histopathological analysis show that oral live *L. plantarum* GKD7 improved subchondral bone architecture, protected articular cartilage against ACLT-induced damage, and reduced synovial inflammation. *L. plantarum* GKD7 also reduced IL-1β and TNF-α production in OA cartilage and synovium. Thus, orally administered live *L. plantarum* GKD7 appears to effectively slow the progression of OA.

## 1. Introduction

Osteoarthritis (OA) is a chronic inflammatory and degenerative joint disease accompanied by often debilitating pain [1]. MRI and micro-CT scans provide vital objective evidence for clinical diagnosis or experimental research of OA-related damage in synovial tissue, cartilage, and subchondral bone [2,3]. NSAIDs and corticosteroids are the mainstays of OA treatment for reducing OA pain, joint swelling, and stiffness [4,5], while joint replacement is considered to be the last therapeutic option for patients with severe joint pain or dysfunction that cannot be treated with pharmacotherapy [4,6]. Importantly, NSAIDs and corticosteroids have undesirable side effect profiles, some of which can be extremely serious [7]. Thus, safer and more efficient treatment strategies are needed for OA.

Reducing proinflammatory cytokine production (e.g., IL-1β and TNF-α are some of the most important therapeutic aims in OA [5], as these inflammatory mediators play important roles in the destructive changes in cartilage and modification to subchondral bone structural changes in OA [8,9]. The secretion of IL-1β and TNF-α from OA synovial fibroblasts and chondrocytes promote the synthesis of proteolytic enzymes that degrade joint extracellular matrices and thus drive OA, worsening the disease-related synovial inflammation, cartilage degeneration, and subchondral bone lesions [9,10,11]. In experimental OA, inhibiting or reducing pro-inflammatory expression suppresses joint degradation, highlighting the importance of such strategies for OA treatment [12,13].

Probiotic *Lactobacillus* spp. strains exhibit a wide variety of beneficial activities in human hosts, including reductions in inflammatory activity [14]. This study investigated the ways in which the *L. plantarum* GKD7 strain affects knee OA in rats subjected to ACLT surgery. Probiotics are considered safe for consumption due to their presence in many foods and their gut defense mechanism [15]. Proinflammatory cytokines such as IL-1β can be reduced by heat-killed *L. plantarum* L-137 in cardiac and adipose tissue in a rat model of metabolic syndrome, while *L. rhamnosus* GG can decrease TNF-α expression in lipopolysaccharide-activated murine macrophages [16,17]. *L. salivarius* UCC118 and *L. plantarum* WCFS1 have shown anti-arthritic activity in mice with collagen-induced arthritis, with evidence of reduced joint destruction and less proinflammatory cytokine activity [18]. Similarly, diverse *Lactobacillus* spp., including *L. rhamnosus* GG and *L. rhamnosus* LR-2, slow the progression of OA by reducing joint pain and inflammation [19,20]. Moreover, much clinical evidence attests to probiotics reducing intestinal damage and inflammation associated with OA disease and probiotics lessen pain severity and cartilage destruction in animal models of OA [19,21,22]. In our previous study, *L. plantarum* GKD7 reduced inflammatory cell infiltration in mice with aspirin-induced gastric injury [23]. We, therefore, speculated that *L. plantarum* GKD7 may slow OA progression by reducing disease-related inflammatory activity. This study examined the effects of orally administered live *L. plantarum* GKD7 upon joint inflammation, pain, swelling, and function in rats with ACLT-induced OA.

## 2. Materials and Methods

### 2.1. Materials

IL-1β antibody (MAB601; final dilution of 1:200) was bought from R&D Systems, Inc. (Minneapolis, MN, USA). TNF-α antibody (A11534; final dilution of 1:200) was bought from ABclonal. Inc. (Woburn, MA, USA). All other chemicals not mentioned above were purchased from Sigma-Aldrich.

### 2.2. Preparation of L. plantarum GKD7

*L. plantarum* GKD7 isolated from Taiwanese pickles was cultured in de Man-Rogosa-Sharpe (MRS) medium for lactobacilli (Merck, Darmstadt, Germany) at 37 °C for 16 h. To prepare the *L. plantarum* GKD7 freeze-dried powder, 0.03% of seed culture was scaled-up in a 15-ton bioreactor with a 12-ton working volume in a culture medium containing 5% glucose, 2% yeast extract, 0.05% MgSO_4_, 0.1% K_2_HPO_4_, and 0.1% Tween 80. After 16 h of incubation at 37 °C, pellets of fermented bacteria were harvested by centrifugation, washed twice with reverse osmosis (RO) water then lyophilized with skim milk. The freeze-dried *L. plantarum* GKD7 powder contained approximately 5 × 10^11^ CFU/g live bacteria, according to the plate count method.

### 2.3. OA Protocol

Eight-week-old male Sprague Dawley rats were supplied by LASCo Inc. (Taipei, Taiwan) and maintained in an animal center under the Institutional Animal Care and Use Committee (IACUC) Guidelines issued by China Medical University (CMU). The rats were randomly assigned to arthrotomy only (controls; *n* = 6), ACLT alone (*n* = 6), or ACLT + *L. plantarum* GKD7 treatment (*n* = 8). ACLT surgery followed the procedures described in previous studies [24,25]. Briefly, the rats were anesthetized with Zoletil 50^®^ (Virbac, Carros, France) before undergoing an arthrotomy to expose the right knee joint, in which the ACL was cut by micro-scissors using surgical loupes. ACLT success was confirmed by the anterior drawer test. Starting from two days after surgery, the daily diet was supplemented with 1 mL RO water in all 3 study groups. *L. plantarum* GKD7 powder was suspended evenly in RO water and administered daily to each individual rat as an oral 100 mg/kg (5 × 10^1^^0^ CFU/kg) dose for 6 weeks in the ACLT + *L. plantarum* GKD7 group; the ACLT-only group and controls were each dosed daily with RO water alone. All rats were sacrificed by CO_2_ on day 49 and the right hind knee samples were collected for micro-CT and pathological analysis, as well as immunohistochemistry (IHC) staining. All experimental procedures were approved by the IACUC of CMU (approval number: CMUIACUC-2021-291).

### 2.4. Weight-Bearing Testing of Hind Paws

The static weight-bearing incapacitance test (Bioseb, Paris, France) was performed every week to assess spontaneous pain and postural deficits. Each rat was placed in an angled plastic chamber and the hind paws were placed on separate sensors to measure between-limb differences in dynamic weight bearing (expressed as g) over a 10-s period [26]. The force value was calculated by the following equation: [Force = weight on left limb − weight on right limb]. Each experiment was repeated 3 times and the mean was recorded for each rat.

### 2.5. Micro-CT Analysis

The right knee joints were collected from the rats after sacrificing with CO_2_ and fixed with 4% paraformaldehyde for micro-CT imaging and analysis [27]. Samples were imaged by a high-resolution micro-CT scanner (Skyscan 2211; Bruker, Kontich, Belgium) under the conditions selected in previous studies [28,29], and InstaRecon^®^ software (Version v.1.3.9.2, Bruker micro-CT, Kontich, Belgium) was used for image reconstruction. Reconstructed cross-sections were reorientated and 59 slices (0.5 mm) were selected, then manual regions of interest (ROI) were drawn following our previous studies [28,29]. The analysis of bone microarchitectural parameters, including BMD (bone mineral density), BMC (bone mineral content), BV/TV (bone volume/tissue volume), BS/TV (bone surface/tissue volume), trabecular thickness (Tb.Th), trabecular separation (Tb.Sp), and trabecular number (Tb.N) were performed by CTAn software (Version 1.18.4, Bruker micro-CT, Kontich, Belgium) following our previous studies [28,29].

### 2.6. Histopathological Analysis

After undergoing micro-CT scanning, the right knee joints were prepared for hamatoxylin and eosin (H&E) staining, Safranin-O/Fast-green staining, or immunohistochemistry (IHC) staining. Briefly, the right knee joints were decalcified by 10% EDTA in phosphate-buffered saline and embedded into paraffin blocks for histology slices with H&E, Safranin-O/Fast-green, and IHC staining. Histopathological changes using an optical microscope, following our previously published procedures [27,30,31,32]. The Osteoarthritis Research Society International (OARSI) histopathology grading system evaluated changes in structural cartilage from the medial tibial plateau (the weight-bearing area) [33,34], defining the grade of damage from 0 to 6 as the depth of OA progression into the cartilage and the stage of damage as the horizontal extent of cartilage damage from 0 to 4. The final score (grade × stage) ranges from 0 (normal cartilage) to 24 points (most advanced grade and most extensive stage) as in previous studies [33,34]. Cartilage degeneration was scored as ‘none’ to ‘severe’ (numerical values 0 to 5) [33,34], and surgery-induced inflammation in the synovial membrane was graded from 0 to 4 as described in our previous studies [35,36].

### 2.7. Statistical Analysis

All data are shown as the mean ± SD and analyzed by using Sigma Plot 12.0 software (Systat Software Inc., Berlin, Germany). The significant difference between the three groups was analyzed by using the unpaired two-tailed Student’s *t*-test and one-way analysis of variance followed by Student-Newman-Keuls post hoc testing. A *p*-value of <0.05 was considered to be statistically significant.

## 3. Results

### 3.1. Oral Live L. plantarum GKD7 Reduces Pain-Related Behavior without Affecting Body Weight

**Figure 1:**
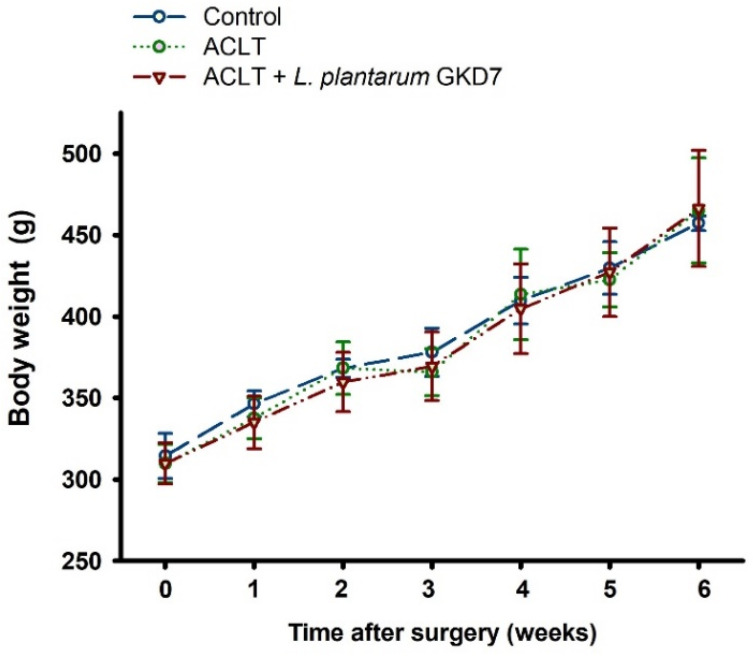
Oral live *L. plantarum* GKD7 did not affect body weight in the OA rat model. Body weights of ACLT rats that did or did not receive oral live administration of live *L. plantarum* GKD7 were not significantly different from those of controls.

**Figure 2:**
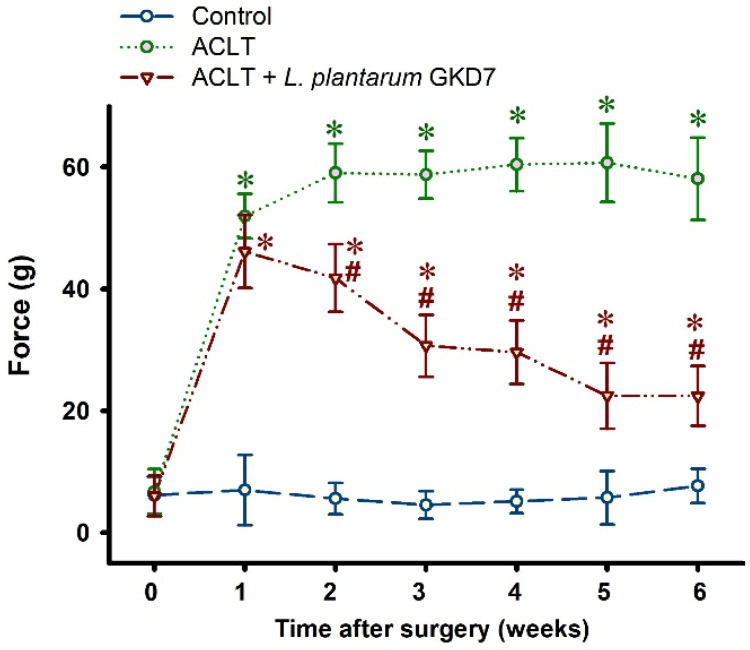
Oral live *L. plantarum* GKD7 improved weight-bearing deficits after ACLT surgery. Daily oral administration of live *L. plantarum* GKD7 was associated with significantly less weight-bearing asymmetry after ACLT surgery compared with ACLT-only rats. * *p* < 0.05 vs. controls; # *p* < 0.05 vs. the ACLT-only group.

By week 6, all 3 study groups had steadily gained body weight from baseline, without any significant between-group differences (Figure 1). At week 1, measurements of asymmetry in weight-bearing posture did not differ significantly between the ACLT-only and ACLT + *L. plantarum* GKD7 groups (52.0 ± 3.7 g vs. 46.1 ± 6.0; *p* = 0.06). From week 1 onwards, ACLT-only rats exhibited severe asymmetry in weight-bearing posture, whereas pain-related behavior in the ACLT + *L. plantarum* GKD7 group was markedly improved from the second week onwards; by week 6, the asymmetry in weight-bearing behavior was approximately half that of the ACLT-only group (22.4 ± 5.0 g vs. 58.1  ±  6.7 g; *p* ≤ 0.05) and close to that of the control group (7.7 ± 2.8 g; *p* < 0.05) (Figure 2).

### 3.2. Micro-CT Analysis Revealed Protective Effects of L. plantarum GKD7 in OA Bone

**Figure 3:**
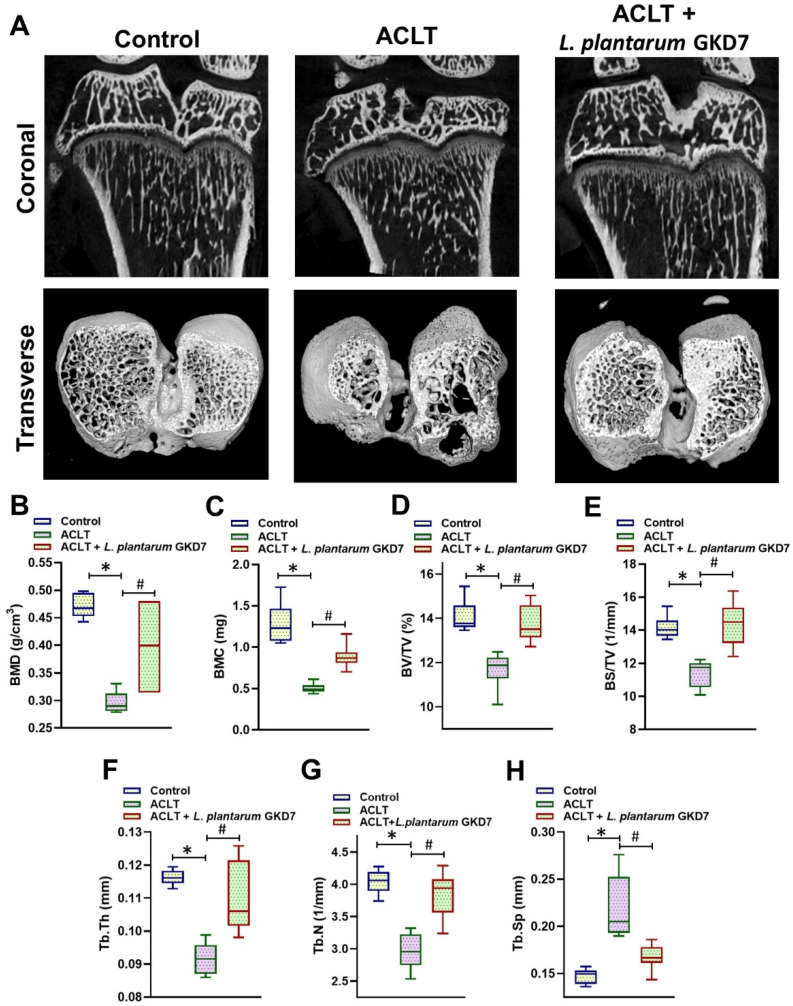
Representative micro-CT images of the right knee joint from rats in the sham-operated group, the ACLT-only group, and the ACLT + *L. plantarum* GKD7 group. (**A**) Representative coronal and transverse micro-CT images of rat knees in each study group. Quantitative analyses of (**B**) BMD, (**C**) BMC, (**D**) BV/TV, (**E**) BS/TV, (**F**) Tb.Th, (**G**) Tb.N, and (**H**) Tb.Sp. * *p* < 0.05 vs. controls; # *p* < 0.05 vs. the ACLT-only group.

Coronal and transverse micro-CT images revealed marked subchondral bone loss in the ACLT-only group compared with controls, whereas minimal loss was evident in the ACLT + *L. plantarum* GKD7-treated rats (Figure 3A). BMD, BMC, BV/TV, BS/TV, Tb.Th, and Tb.N measurements were all significantly smaller, while Tb.Sp measurements were significantly larger, in the ACLT-only group compared with controls (Figure 3B–H). In contrast, daily oral administration of live *L. plantarum* GKD7 was associated with significant improvements in changes to subchondral bone induced by ACLT surgery (Figure 3B–H). Thus, *L. plantarum* GKD7 appears to reduce deleterious changes to knee joint structure induced by ACLT.

### 3.3. Histopathological Analysis of L. plantarum GKD7 in OA Rats

**Figure 4:**
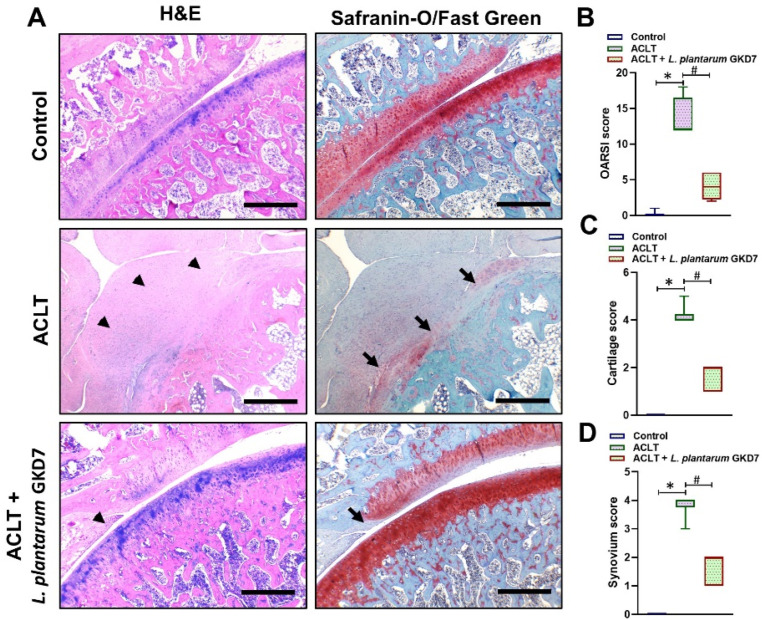
Histological evidence. (**A**) The images show representative knee joints from each group, consisting of coronal sections of articular cartilage stained with H&E and Safranin-O/Fast Green (magnification 5×). The arrowheads denote synovial hyperplasia and the arrows denote cartilage damage. Quantitative analyses of (**B**) OARSI scores, (**C**) cartilage scores, and (**D**) synovium scores. Scale bar = 500 µm. * *p* < 0.05 vs. controls; # *p* < 0.05 vs. the ACLT-only group.

Staining by H&E and Safranin-O/Fast Green revealed less injury (imaged were more intact) in articular cartilage samples from the controls and ACLT + *L. plantarum* GKD7 group compared with the minimal cartilage in the ACLT-only samples (Figure 4A). ACLT-induced synovial inflammation (marked by arrowheads) and cartilage damage (arrows) were minimal in the ACLT + *L. plantarum* GKD7 samples, but very apparent in the ACLT-only samples (Figure 4A). OARSI, cartilage, and synovium scores were significantly superior in the ACLT + *L. plantarum* GKD7 group compared with scores in the ACLT-only group, while the ACLT-only scores were significantly superior compared with the control group (Figure 4B–D).

### 3.4. IHC Analysis of Proinflammatory Markers

**Figure 5:**
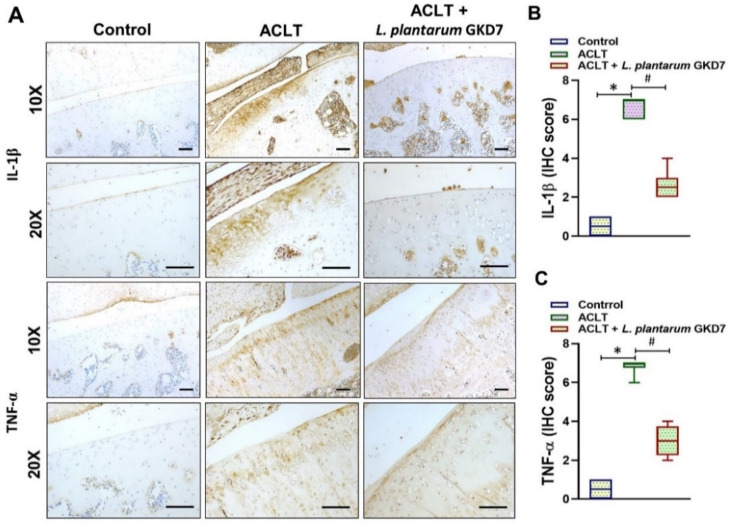
Oral live *L. plantarum* GKD7 reduced key proinflammatory cytokines in OA cartilage. (**A**) IHC staining of IL-1β and TNF-α expression in representative cartilage from controls, ACLT-only rats, and ACLT + *L. plantarum* GKD7 rats. Quantitative analyses of (**B**) IL-1β and (**C**) TNF-α in cartilage. Scale bar = 100 µm. * *p* < 0.05 vs. controls; # *p* < 0.05 vs. the ACLT-only group.

**Figure 6:**
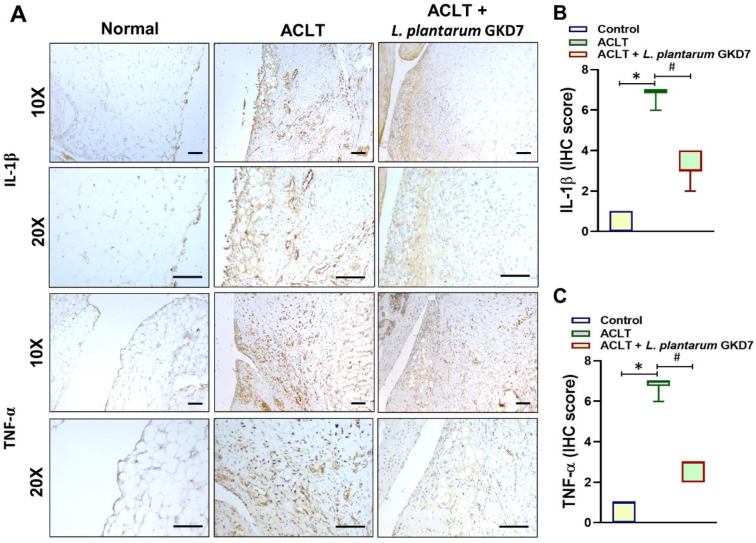
Oral live *L. plantarum* GKD7 reduced levels of TNF-α and IL-1β expression in OA synovium. (**A**) IHC staining of IL-1β and TNF-α in representative synovium from controls, ACLT-only rats, and ACLT + *L. plantarum* GKD7 rats. Quantitative analyses of (**B**) IL-1β and (**C**) TNF-α in synovium. Scale bar = 100 µm. * *p* < 0.05 vs. controls; # *p* < 0.05 vs. the ACLT-only group.

As shown in Figure 5A and Figure 6A, IL-1β and TNF-α levels were markedly upregulated in ACLT-only cartilage and synovium compared with control and *L. plantarum* GKD7 cartilage and synovium. The ACLT-only group had significantly higher IHC scores of cartilage and synovium compared with both the controls and the *L. plantarum* GKD7 group; these values were significantly decreased by *L. plantarum* GKD7 when compared with ACLT-only (Figure 5B,C and Figure 6B,C).

## 4. Discussion

Proinflammatory cytokines have an important role in OA joint destruction [8,37], promoting damage in joint extracellular matrices and stimulating the progression of OA [9,10]. High IL-1β and TNF-α levels upregulate proteolytic enzymes, such as MMPs and ADAMTS family, which degrade the extracellular matrix [9,11]. Probiotics are well recognized for their anti-inflammatory properties [38,39] and previous research has highlighted the IL-1β and TNF-α cytokines as important targets in probiotic treatment strategies for arthritic diseases [19,40]. In particular, consumption of *Lactobacillus* spp. reduces proinflammatory cytokine production in experimental OA [19,41,42]. This was supported by our study results, as IL-1β and TNF-α levels in rat cartilage and synovium were markedly reduced by daily oral administration of live *L. plantarum* GKD7.

Coronal and transverse micro-CT imaging revealed minimal changes in subchondral bone architecture in the ACLT + *L. plantarum* GKD7 group, whereas there was marked subchondral bone loss in the ACLT-only group compared with controls. Subchondral bone lesions, including bone marrow edema and angiogenesis, are known to contribute to OA joint destruction [43,44], while IL-1β and TNF-α stimulate MMPs and ADAMTSs activity that subsequently worsens bone architecture, reflected by reductions in BMD, BMC and BV values, which promote OA disease progression [9,45]. The recent studies also indicated that subchondral bone lesions, including bone marrow edema and angiogenesis, also contribute to the OA joint destruction. Our micro-CT images revealed significant improvements in bone microarchitectural parameters of rats treated with live *L. plantarum* GKD7 following ACLT surgery compared with ACLT-only rats. In the *L. plantarum* GKD7 group, we observed significantly lower cartilage and synovium IHC scores, and also significantly reduced IL-1β and TNF-α levels, compared with the ACLT-only group. We, therefore, speculate that *L. plantarum* GKD7 ameliorates OA-induced changes in bone microarchitectural parameters by inhibiting proinflammatory levels in cartilage and synovial tissue. Thus, our data suggest that daily oral administration of live *L. plantarum* GKD7 can effectively reduce synovial inflammation and damage to bone architecture induced by ACLT surgery.

OA disease is accompanied by often severe pain [46]. In OA, the upregulation of TNF-α and IL-1β expression in the injured joint induces mechanical pain hypersensitivity by increasing the expression of nerve growth factor, which in turn increases the phosphorylation state of transient receptor potential vanilloid receptor 1 (TRPV1) [46,47,48], critical mediators of inflammatory pain signaling. Moreover, OA-induced disturbance of subchondral bone and cartilage destruction results in abnormal load-bearing in the joints and is an important contributor to chronic pain during non-weight-bearing activities [44,49]. This abnormal load-bearing causes the release of multiple signaling mediators such as nerve growth factor, neuropeptide substance P, and neurokinin-1 in the osteochondral area, resulting in persistent nociception stimulation and the characteristic OA pain [44]. Thus, reducing the expression of TNF-α and IL-1β and improving the cartilage destruction and “abnormal” loading within the subchondral bone are important targets of OA treatment [1,43,45]. In our results, ACLT surgery significantly increased levels of TNF-α and IL-1β expression, led to the development of weight-bearing deficits in rat hind legs, and structural damage in articular cartilage, compared with changes observed in the sham-operated group. Daily oral administration of live *L. plantarum* GKD7 significantly reduced these ACLT-induced effects, which suggests that this probiotic strain can effectively ameliorate OA pain.

## 5. Conclusions

Consumption of live *L. plantarum* GKD7 may improve OA disease progression and associated joint pain by reducing levels of IL-1β and TNF-α in OA joints and by improving weight-bearing asymmetry. We suggest that *L. plantarum* GKD7 may be a useful strategy for improving the signs and symptoms of OA.

## Data Availability

The raw data for this study are available from the corresponding authors.
